# Biophysical and structural mechanisms of epilepsy-associated mutations in the S4-S5 Linker of KCNQ2 channels

**DOI:** 10.1080/19336950.2025.2464735

**Published:** 2025-02-19

**Authors:** Inn-Chi Lee, Yen-Yu Yang, Hsueh-Kai Chang, Swee-Hee Wong, Shi-Bing Yang

**Affiliations:** a Institute of Medicine, School of Medicine, Chung Shan Medical University, Taichung, Taiwan; b Division of Pediatric Neurology, Department of Pediatrics, Chung Shan Medical University Hospital, Taichung, Taiwan; c Thermo Fisher Scientific, San Jose, CA, USA; d Institute of Biomedical Sciences, Academia Sinica, Taipei, Taiwan

**Keywords:** KCNQ, channelopathy, developmental and epileptic encephalopathy, self-limited familial neonatal epilepsy, gating

## Abstract

Mutations in *KCNQ2* are linked to various neurological disorders, including neonatal-onset epilepsy. The severity of these conditions often correlates with the mutation’s location and the biochemical properties of the altered amino acid side chains. Two mutations affecting aspartate at position 212 (D212) in the S4-S5 linker of KCNQ2 have been identified. Interestingly, while the charge-conserved D212E mutation leads to severe neonatal-onset developmental and epileptic encephalopathy (DEE), the more dramatic substitution to glycine (D212G) results in self-limited familial neonatal epilepsy (SLFNE), a much milder pathology. To elucidate the underlying mechanisms, we performed electrophysiological studies and *in silico* simulations to investigate these mutations’ biophysical and structural effects. Our findings reveal that the D212E mutation stabilizes the channel in the voltage sensor down-state and destabilizes the up-state, leading to a rightward shift in the voltage-dependent activation curve, slower activation kinetics, and accelerated deactivation kinetics. This disruption in KCNQ2 voltage sensitivity persists even in the more physiologically relevant KCNQ2/3 heterotetrameric channels. In contrast, the D212G mutation primarily destabilizes the up-state, but its impact on voltage sensitivity is significantly reduced in KCNQ2/3 heterotetrameric channels. These findings provide key insights into the biophysical and structural basis of *KCNQ2* D212 mutations and their contribution to epilepsy-related symptoms, offering a clearer understanding of how these mutations drive the varied clinical outcomes observed in patients.

## Introduction

KCNQ2 is a voltage-gated potassium channel primarily expressed in the central nervous system, where it plays a key role in regulating neuronal excitability and is often co-expressed with KCNQ3 channels [1,2]. Mutations in *KCNQ2* are frequently associated with neonatal epilepsy, presenting in two main forms: self-limited familial neonatal epilepsy (SLFNE) and neonatal-onset developmental and epileptic encephalopathy (DEE) [3–6]. SLFNE typically manifests in the neonatal period, followed by a relatively benign course, with most individuals experiencing seizure remission within the first year of life and normal neurodevelopment afterward [7,8]. In contrast, *KCNQ2*-associated neonatal-onset DEE is more severe, characterized by frequent, often drug-resistant seizures that persist throughout life, leading to poor neurodevelopmental outcomes [5,9,10]. Despite advances in genetic testing and diagnosis, no effective treatments currently exist that can improve long-term neurodevelopmental outcomes in *KCNQ2-*associated neonatal-onset DEE, highlighting the need to explore the underlying mechanisms of these mutations.

Each KCNQ2 subunit consists of six transmembrane segments (S1–S6), where the S1–S4 segments form the voltage-sensing domain responsible for detecting changes in membrane potential [11]. The S4 segment functions as the primary voltage sensor due to its positively charged arginine residues, which are responsible for detecting transmembrane voltage changes [12]. At resting membrane potential, the voltage sensor resides in the down-state, oriented toward the inner leaflet of the lipid bilayer [13,14]. Upon membrane depolarization, the voltage sensor shifts to the up-state, moving toward the extracellular leaflet. The S5 and S6 segments form the pore domain, with the loop between them containing a highly conserved selectivity filter critical for potassium ion selectivity [15]. The pore domain is mechanically coupled to the voltage-sensing domain, and its opening is regulated by intracellular signaling molecules, such as PIP2, which can only engage when the voltage sensor is in the permissive up-state [15–17]. A unique feature of KCNQ channels is their domain-swapping architecture, in which the voltage-sensing domain of one subunit interacts with the pore domain of an adjacent subunit [18]. This arrangement ensures tight coupling between voltage sensor movements and pore gating, making the channel highly sensitive to the membrane lipid signaling pathways [17,19]. Mutations in the voltage-sensing domain or near the PIP2-binding region can lead to either DEE or SLFNE [3,16]. KCNQ2 channels typically form tetrameric complexes, either as homotetramers or heterotetramers. A well-known example is the KCNQ2/KCNQ3 heterotetramer, which predominates in the adult central nervous system and is crucial in maintaining neuronal excitability and preventing hyperexcitability associated with epilepsy [20,21].

Most DEE-associated *KCNQ2* mutations are loss-of-function mutations that severely impair channel gating or reduce surface expression, leading to profound clinical symptoms [3]. These mutations often lead to physiochemical alterations of side chain properties, such as changes in hydrophobicity, charge, or size [22–24]. In contrast, SLFNE-associated mutations are typically more conservative, preserving key properties like charge or hydrophobicity and leading to milder clinical presentations [3,24,25]. The location of the mutation within the KCNQ2 channel is also a critical factor in determining the severity of the phenotype. Nonsense, splice and frameshift mutations result in SLFNE due to haploinsufficiency [26]. However, DEE mutations are often missense mutations with a dominant negative effect, typically located in key regions such as the voltage sensing or pore domains. These are critical domains for channel function and are less tolerant of subtle alterations [27].

Recent studies have identified mutations at aspartate residue 212 (D212) in the S4-S5 linker region of KCNQ2 that result in diverse clinical outcomes. Interestingly, the D212E mutation, where the charge-conserved glutamate replaces aspartate, leads to DEE despite the minimal physiochemical change – a difference of only one carbon atom between the side chains [28]. In contrast, the D212G mutation, where the much smaller, neutral glycine replaces aspartate, results in SLFNE, which is associated with relatively milder symptoms [29]. To elucidate the molecular and physiochemical mechanisms underlying these contrasting clinical outcomes, we assessed the effects of the D212E and D212G mutations on Kv7.2 currents expressed in HEK293 cells and performed *in silico* modeling to evaluate their structural impacts on the KCNQ2 channel. Our findings reveal that the D212E mutation drastically disrupts the conformational transition between the down- and up-states of the S4-S5 linker, which couples the voltage sensor to the pore domain. In contrast, the D212G mutation moderately impacts the S4-S5 linker’s conformational changes. These results provide insight into the varying clinical outcomes associated with these mutations and underscore the critical role of the D212 residue in maintaining the mechanical coupling between the voltage sensor and the pore domain during voltage-dependent channel gating.

## Results

**Figure 1.**
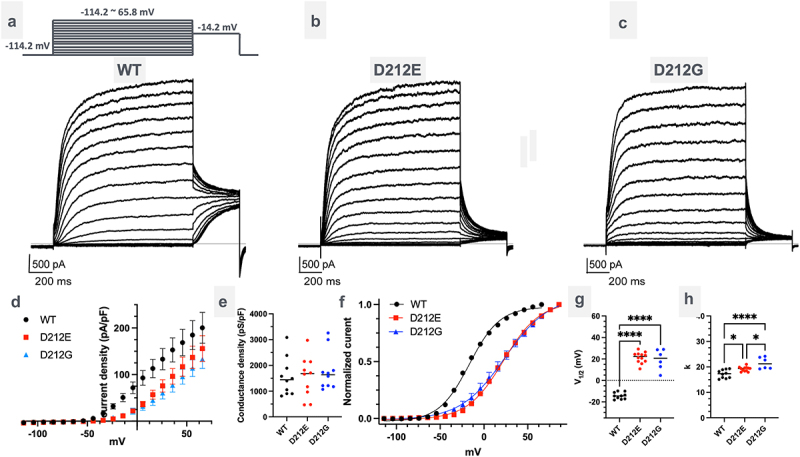
Functional characterization of dee-causing *KCNQ2*-D212E and SLFNE-causing *KCNQ2*-D212G mutations. (a-c) Representative current traces of human KCNQ2 channels in HEK293 cells transfected with KCNQ2 WT (a), *KCNQ2*-D212E (b), or *KCNQ2*-D212G (c). Cells were held at −114.2 mV and subjected to 3-second voltage steps ranging from −114.2 mV to 65.8 mV in 10 mV increments, followed by a return to −14.2 mV. (d) Steady-state current densities measured at the end of the 3-second voltage steps plotted against test voltage. *KCNQ2*-D212E and *KCNQ2*-D212G were activated at more depolarized potentials than WT. (e) Normalized conductance densities measured between 55.8 and 65.8 mV showed comparable current densities among WT, *KCNQ2*-D212E, and *KCNQ2*-D212G (*n* = 10, 10, and 10 for WT, D212E, and D212G, respectively; one-way ANOVA). (f-h) Activation curves of WT, *KCNQ2*-D212E, and *KCNQ2*-D212G reveal rightward shifts for both mutants, indicating elevated V_1/2_ values (g) (eq.1) (*n* = 10, 12, and 6 for WT, D212E, and D212G, respectively; *p* < 0.001 for WT vs D212E and *p* < 0.001 for WT vs D212G; one-way ANOVA). Both *KCNQ2*-D212E and *KCNQ2*-D212G exhibited increased k values, suggesting impaired voltage sensing (H) (*n* = 10, 12, and 6 for WT, D212E, and D212G, respectively; *p* < 0.05 for WT vs D212E, *p* < 0.05 for D212E vs D212G, and *p* < 0.001 for WT vs D212G; one-way ANOVA).

To investigate the functional consequences of the D212E mutation in the S4-S5 linker of KCNQ2, we performed whole-cell patch-clamp recordings in HEK293 cells transfected with either wild-type (WT), *KCNQ2*-D212E or *KCNQ2*-D212E plasmids. Voltage-step protocols were applied, and the resulting current-voltage relationships were analyzed. Compared to WT KCNQ2, both KCNQ2-D212E and KCNQ2-D212G channels exhibited a significant rightward shift in the voltage dependence of activation, indicating that stronger depolarization is required to activate KCNQ2-D212E and KCNQ2-D212G (Figure 1a–d). Notably, at more depolarized voltages, the current densities of KCNQ2-D212E KCNQ2-D212G and WT channels were comparable (Figure 1e and Table 1), suggesting that the mutation affects voltage sensitivity without altering maximum conductance.

**Table 1.** The biophysical parameters of the KCNQ2 mutations.

	V_1/2 (mV)_ (mean±SD)	*k* (Slope) (mV/e) (mean±SD)	Conductance densities (pS/pF) (mean±SD)
KCNQ2 WT (2 ug)†	−14.2 + 3.1 (*n* = 10)	17.2 + 1.6 (*n* = 10)	45.6 + 5.6 (*n* = 10)
D212E (2 ug)	21.4 + 5.1**** (*n* = 12)	19.2 + 0.9* (*n* = 12)	41.5 + 5.6 (*n* = 10)
D212G (2 ug)	19.4 + 9.6**** (*n* = 6)	21.5 + 2.3**** (*n* = 6)	39.3 + 11.3 (*n* = 10)
KCNQ2 WT+KCNQ3 WT (1ug:1 ug)	−27.9 + 8.6 (*n* = 14)	17.6 + 4.2 (*n* = 14)	
KCNQ2 WT+D212E+KCNQ3 WT (0.5 ug: 0.5 ug: 1 ug)	−17.9 + 10* (*n* = 14)	17.9 + 2.8 (*n* = 14)	
KCNQ2 WT+D212G+KCNQ3 WT (0.5 ug: 0.5 ug: 1 ug)	−25.5 + 9.9 (*n* = 7)	22.3 + 5* (*n* = 7)	

*indicates *p* < 0.05; **, ****, *p* < 0.001. One-way ANOVA.

WT, wild type; V is the test potential; V½, half-maximal activation voltage; SD, standard deviation.

To determine whether the reduced current density observed in KCNQ2-D212E and KCNQ2-D212G was due to impaired voltage-dependent activation, we constructed the voltage-dependent activation curves for WT, KCNQ2-D212E, and KCNQ2-D212G. Both mutant channels exhibited a rightward shift in their activation curves (Figure 1f). By fitting the voltage-dependent activation curves to the Boltzmann function (eq.1), we observed a significant shift in V_1/2_ for both mutants (Figure 1g and Table 1). Interestingly, in addition to the V_1/2_ shift, both mutants exhibited an increased *k* value, indicating impaired voltage sensing (Figure 1h and Table 1).

**Figure 2.**
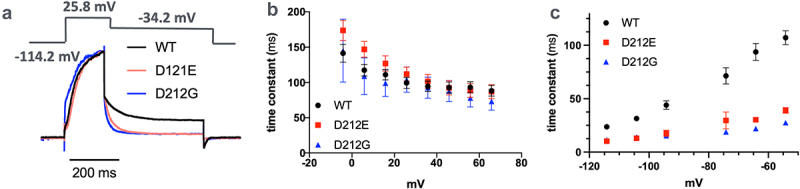
*KCNQ2*-D212E and *KCNQ2*-D212G accelerate *KCNQ2* channel closure. (a) Representative normalized current traces from HEK293 cells transfected with *KCNQ2* WT (black), *KCNQ2*-D212E (red), and *KCNQ2*-D212G (blue). After depolarization to 25.8 mV, the voltage was stepped back to −34.2 mV. (b) The activation time constant (τ) plotted against test voltage pulses shows that neither *KCNQ2*-D212E nor *KCNQ2*-D212G affected KCNQ2 channel activation (*n* = 10, 11, and 5 for WT, D212E, and D212G, respectively; Two-way ANOVA). (c) Both KCNQ2-D212E and KCNQ2-D212G significantly accelerated channel deactivation across all test voltages (eq.2)(*n* = 10, 10, and 6 for WT, D212E, and D212G, respectively; Two; *p* < 0.001 for WT vs D212E, *p* < 0.001 for WT vs D212G; one-way ANOVA).

To further characterize the gating properties, we analyzed both the activation and deactivation kinetics of WT, KCNQ2-D212E and KCNQ2-D212E channels (Figure 2a–c) using previously established quantification methods(eq.2) [30]. We found that both mutants had minimal effects on *KCNQ2* channel activation kinetics (Figure 2b). However, upon stepping back to hyperpolarized potentials, both KCNQ2-D212E and KCNQ2-D212G channels closed more rapidly than WT channels (Figure 2c), suggesting that both mutants accelerate the decoupling of voltage-sensor to the pore domain in the down-state.

**Figure 3.**
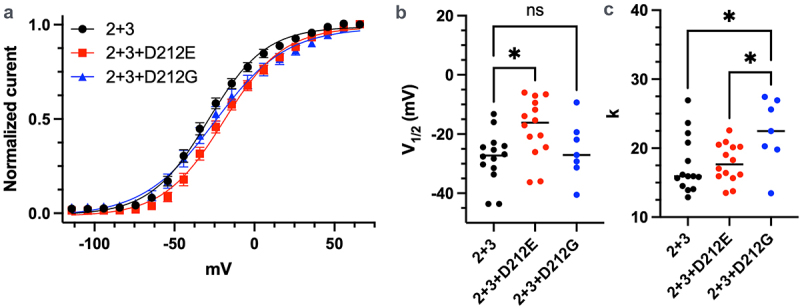
Dee-causing *KCNQ2*-D212E mutation moderately affects *KCNQ2/3* heterotetrameric channels. (a) Activation curves of heterotetrameric *KCNQ2/3* channels containing WT-*KCNQ2*, *KCNQ2*-D212E, or *KCNQ2*-D212G. *KCNQ2*-D212E-containing *KCNQ2/3* heterotetrameric channels exhibited moderately impaired activation, as indicated by a rightward shift in the activation curve. In contrast, *KCNQ2*-D212G-containing *KCNQ2/3* heterotetrameric channels were comparable to WT, with a slightly flattened activation curve at more depolarized voltages (*n* = 14, 14, and 7 for WT, D212E, and D212G, respectively). (b) Elevated V_1/2_ values were observed in *KCNQ2*-D212E-containing *KCNQ2/3* heterotetrameric channels compared to WT (*n* = 14, 14, and 7 for WT, D212E, and D212G, respectively; *p* < 0.05, one-way ANOVA). (c) *KCNQ2*-D212G-containing *KCNQ2/3* heterotetrameric channels exhibited increased *k* values, indicating impaired voltage sensing (*n* = 14, 14, and 7 for WT, D212E, and D212G, respectively; *p* < 0.05 for WT vs. D212E and D212E vs. D212G, one-way ANOVA).

Patients carrying the *KCNQ2*-D212E mutation develop DEE, while patients with the KCNQ2-D212G mutation develop SLFNE, which presents with milder and transient neurological symptoms. Surprisingly, despite glycine (D212G) being smaller and uncharged – expected to disrupt channel function more significantly than glutamate (D212E) – patients with the *KCNQ2*-D212G mutation exhibit a much milder phenotype than those with *KCNQ2*-D212E. Given that M-currents in the adult central nervous system are predominantly mediated by KCNQ2/3 heterotetrameric channels, we cotransfected HEK293 cells with WT-*KCNQ2, KCNQ2*-D212E, or *KCNQ2*-D212G together with *KCNQ3* at a 1:1:2 ratio, to reflect the stoichiometry of KCNQ2/3 heterotetramers typically found in the adult central nervous system, where two KCNQ2 subunits and two KCNQ3 subunits form the functional channel complex. Surprisingly, the *KCNQ2*-D212E mutation caused a stronger rightward shift in the activation curve and had a pronounced effect on the voltage-dependent activation of KCNQ2/3 heterotetrameric channels (Figure 3a). This discrepancy was particularly evident at physiological membrane potentials (−50 to 0 mV), where the KCNQ2/3-D212E channel availability was reduced to only 50–80% of WT KCNQ2/3 or KCNQ2/3-D212G. By fitting the voltage-dependent activation curves (Eq.1), we found that heterotetrameric channels containing *KCNQ2*-D212E had a significant shift in V_1/2_ (Figure 3b and Table 1). Interestingly, only heterotetrameric channels containing *KCNQ2*-D212G, but not *KCNQ2*-D212E, exhibited an increased *k* value (Figure 3c and Table 1), suggesting a difference in the voltage-sensing mechanism between the two mutants.

**Figure 4.**
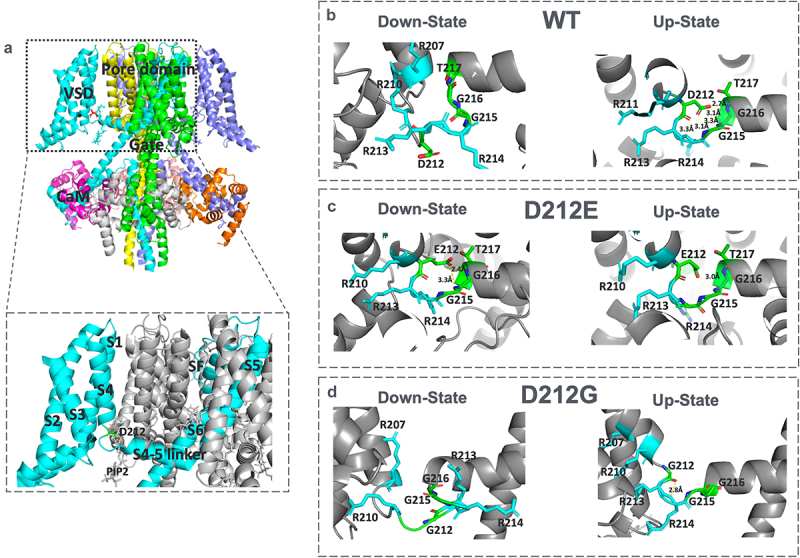
Predicted structures of WT, *KCNQ2*-D212E, and *KCNQ2*-D212G channels. (A) Side view of the predicted WT *KCNQ2* channel structure. The voltage-sensing domain and the S4-S5 linker region, part of the pore domain, are highlighted in the boxed area. (B – D) Close-up views of the S4-S5 linker region: (B) for WT *KCNQ2*, in the down-state, the region containing D212 forms a loosely packed, unstructured loop. In the up-state, the S4-S5 linker stretches, and the carboxyl group of D212 forms up to five potential hydrogen bonds with the peptide backbone of G215 and G216. (C) For *KCNQ2*-D212E, in the down-state, the carboxyl group of glutamate forms two hydrogen bonds with G215 and G216, stabilizing the closed conformation. In the up-state, the bulkier glutamate side chain bends inward, allowing only one hydrogen bond with G215, resulting in a less stable conformation. (D) For KCNQ2-D212G, in the down-state, the region forms a loosely packed loop without hydrogen bonds, resembling the WT KCNQ2 in the down-state. In the up-state, the smaller glycine residue forms only one potential hydrogen bond with G215, leading to a less stable conformation compared to WT.

Given the relatively conservative nature of the D212E mutation, where glutamate replaces aspartate (both negatively charged), the significant alterations in gating properties were unexpected. To explore the structural basis of these functional changes, we used *in silico* modeling of both WT and KCNQ2-D212E channels based on existing KCNQ channel structures (Figure 4a). Up to date, all the available KCNQ2 structures have been determined at zero voltage, presumably representing the voltage sensor in the up-state [11,12,31]. Since KCNQ2 with the voltage sensor in the down-state is structurally undetermined, we modeled both closed (down-state) and open (up-state) conformations based on KCNQ1, which shares a highly conserved S4-S5 linker region.

Similar to KCNQ1, the S4-S5 linker in KCNQ2 formed a loosely packed loop lacking a well-defined secondary structure in the down-state (Figure 4b). Upon depolarization, the voltage sensor moved to the up-state, stretching the S4-S5 linker and inducing the formation of an additional alpha-helix (Figure 4b). In this conformation, 5 potential binding partners of the D212 carboxyl group are located on the peptide backbone of G215 and G216 are within the range (<3.5 Å) and a hydrogen bond is very likely being formed [32]. With the help of a hydrogen bond, a stable mechanical coupling between the voltage sensor and the pore domain can be maintained at the up-state.

In contrast, the *KCNQ2*-D212E mutation introduced distinct structural changes in the S4-S5 linker between the down- and up-states. In the down-state, the carboxyl group of D212E could form a hydrogen bond with G215 or G216, stabilizing the down-state (Figure 4c). However, in the up-state, the larger side chain of glutamate bent inward. The only plausible hydrogen bond formed with the amine backbone of the G215 (Figure 4c) results in a much less stable coupling between the voltage sensor and the pore domain at the up-state. This destabilization likely explains the rapid deactivation (Figure 2c) in KCNQ2-D212E, as the channels require higher voltage to activate and maintain the open conformation (Figure 1g and Table 1).

To understand this discrepancy, we also generated structural models of KCNQ2-D212G. In the down-state, the S4-S5 linker in KCNQ2-D212G resembled WT, forming a loosely packed loop without a stable secondary structure (Figure 4d). In the up-state, the backbone of glycine at position 212 could form two hydrogen bonds with only G215 or G216 (Figure 4d), leading to less stable coupling between the voltage sensor and the pore domain, which might also lead to the rapid deactivation in KCNQ2-D212G (Figure 2c).

## Discussion

**Figure 5.**
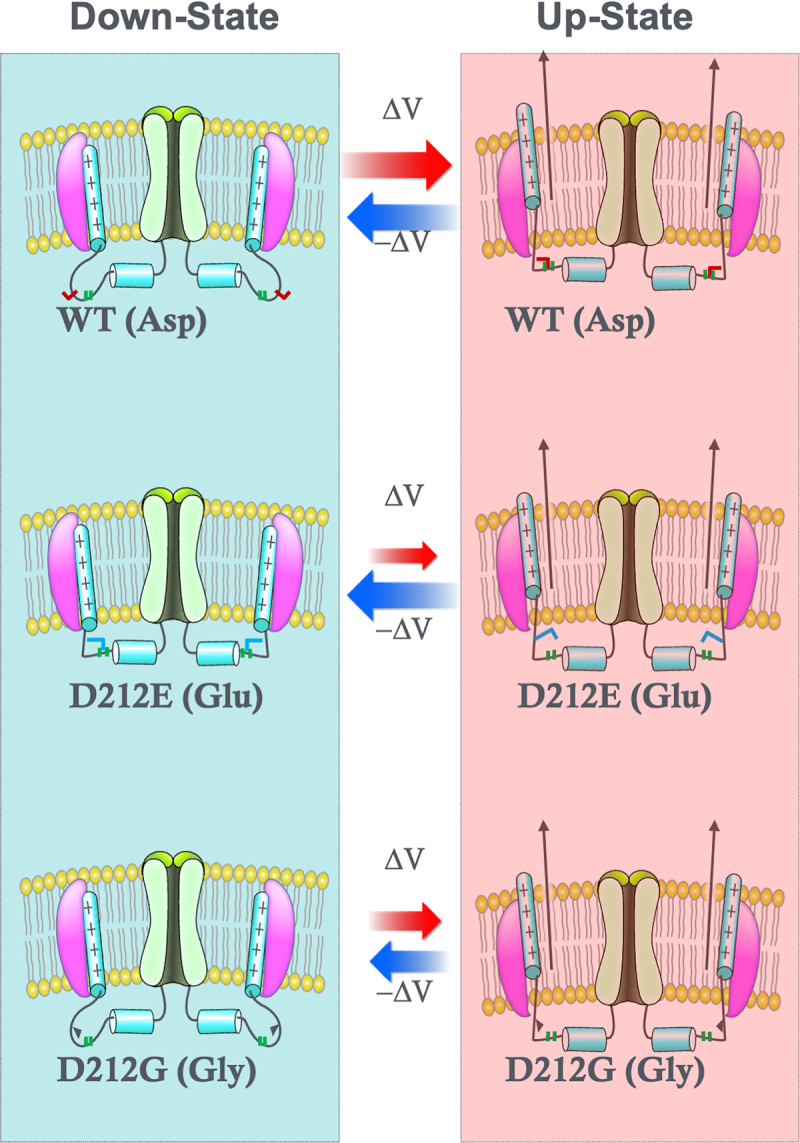
Schematic representation of D212 in voltage-dependent gating of *KCNQ2* channels. In the voltage-sensor down-state, the S4-S5 linker forms an unstructured loop, and D212 does not establish stable bonds with potential interacting partners. Upon depolarization, the voltage sensor moves upward, restructuring the S4-S5 linker. In the up-state, the S4-S5 linker stretches, allowing D212 to form stable bonds with multiple partners in the linker region. For the dee-causing D212E mutation, the glutamate residue stabilizes the down-state by creating a stable structure with nearby partners. However, in the up-state, the bulkier glutamate side chain prevents stable bond formation, destabilizing the up-state conformation. For the slfne-causing D212G mutation, the glycine residue is too small to interact effectively with potential binding partners, resulting in a less stable down-state. In the up-state, the short glycine residue similarly fails to form stable bonds with the S4-S5 linker, reducing the stability of the voltage-sensor up-state.

In this study, we demonstrated that the aspartate residue at the position 212 in the S4-S5 linker of KCNQ2 plays a critical role in regulating the coupling between the voltage sensor and the pore domain. Our findings suggest that the size of the side chain, rather than its charge, plays a more critical role in determining the effect of mutations on KCNQ2 voltage sensing (Figure 5). This observation aligns with the clinical outcomes: patients carrying the *KCNQ2*-D212E mutation developed DEE, whereas those with the *KCNQ2*-D212G mutation exhibited the milder SLFNE phenotype (Figure 3) [28,29].

The D212E variant was not observed in approximately 6,500 individuals of European or African-American ancestry in the NHLBI Exome Sequencing Project or the *KCNQ*2/3 Epileptic Encephalopathy Database (rikee.org) [3]. To date, there has been only one reported case of DEE associated with the *KCNQ2*-D212E mutation [28]. In our study, we confirmed that the D212E mutation reduced the voltage sensitivity of KCNQ2 channels, highlighting its significant impact on channel gating. DEE-associated mutations often cluster in critical domains, such as the voltage-sensing domain or selectivity filter, while SLFNE mutations are more widely spread. Interestingly, D212 is located in the S4-S5 linker, a region not previously linked to DEE, possibly due to its short length [3].

Recent structural studies of KCNQ1, particularly in its down-state conformation, provide valuable insights into the role of the S4-S5 linker in voltage-gated potassium channels. In the down-state, the S4-S5 linker appears flexible and unstructured immediately after the S4 voltage sensor. However, during depolarization, as the voltage sensor transitions to the up-state, the linker stretches and forms an additional alpha-helical turn, which could stabilize mechanical coupling between the voltage sensor and the pore domain. This conformational change likely improves channel gating efficiency by translating voltage sensor movements into pore openings [13]. We developed a gating model to illustrate the critical role of this D212 residue, and our modeling of KCNQ2, based on the KCNQ1 structure, suggests that the D212 region similarly forms a large, unstructured loop in the down-state (Figure 4a,b). In the up-state, D212 helps stabilize the channel by hydrogen bond that can be formed with multiple neighboring amino acids in the S4-S5 linker (Figure 4b). However, when D212 is mutated to glutamate, the bulkier side chain allows for hydrogen bonding in the down-state but bends inward in the up-state, preventing stable hydrogen bond formation with the S4-S5 linker (Figure 4c). In contrast, mutation to glycine, with its smaller and more flexible side chain, results in an unstructured loop in the down-state but fails to form a stable structure in the up-state (Figure 4d). This flexibility helps partially preserve channel functionality, placing the D212G mutation’s voltage sensitivity and resulting clinical phenotype in-between the up-state favored WT and the down-state stable D212E. Lastly, although the S4-S5 linker forms part of the PIP2 binding pocket [15–17], D212 is unlikely to directly regulate KCNQ2 gating via interaction with PIP2, as predicted and solved structures place D212 at least 6 Å away from the PIP2 molecule. However, we cannot entirely rule out the possibility of D212 interacting with PIP2 during large conformational transitions, as such interactions could occur during gating.

Our electrophysiology results strongly support our structural predictions, demonstrating that the *KCNQ2*-D212E mutant exhibits altered channel gating properties, consistent with a shift in the equilibrium toward the down-state. Specifically, the *KCNQ2*-D212E mutant required a higher voltage to activate, reflecting a rightward shift in the activation curve (Figure 1 and Table 1). This indicates that the mutation destabilizes the voltage-sensor to pore domain coupling in the up-state, making it more difficult for the channel to open under normal physiological conditions. Conversely, the faster deactivation kinetics imply that the coupling between the voltage sensor and pore domain rapidly disassembles upon membrane repolarization, reinforcing the hypothesis that the channel’s overall conformational equilibrium is shifted toward the down-state (Figures 1, 2 and Table 1). Together, these findings suggest that the D212E mutation destabilizes the voltage-sensor and pore domain coupling in the up-state, but forms a stable structure when the voltage sensor is in the down-state (Figure 4c). This alteration in voltage sensitivity persists even in heterotetrameric KCNQ2/3 + KCNQ2-D212E channels (Figure 3 and Table 1), which aligns with the severe clinical manifestations observed in DEE patients carrying the D212E mutation.

Interestingly, a similar phenomenon is observed in *KCNQ1*-D242N mutations found in patients with long QT syndrome. The KCNQ1-D242 residue is homologous to KCNQ2-D212, and patients with KCNQ1-D242N show prolonged RR intervals and electrophysiology studies due to impaired I_KS_ currents [33]. While previous studies suggested that the *KCNQ1*-D242N mutation disrupts coupling between the voltage sensor and positively charged arginine residues in the S4-S5 linker, our findings suggest that the mechanisms might be more similar to those in KCNQ2, with conformational shifts playing a key role. These results highlight the importance of the size and chemical properties of mutated residues in determining clinical outcomes rather than just the magnitude of the alteration [3,33].

An intriguing aspect of our study is the effect of the *KCNQ2*-D212E and *KCNQ2*-D212G mutations on the physiologically relevant KCNQ2/3 heterotetramer. Our results show that, although both *KCNQ2*-D212E and *KCNQ2*-D212G significantly affect voltage sensing in homotetrameric KCNQ2 channels, only *KCNQ2*-D212E, not *KCNQ2*-D212G, impairs voltage sensing in heterotetrameric KCNQ2/3 channels (Figures 1, 3, and Table 1). Interestingly, the *KCNQ2*-D212G mutation has little to no effect on voltage sensing in heterotetrameric KCNQ2/3 channels, despite its substantial impact on homotetrameric KCNQ2 channels (Figure 3). This result contrasts with findings from Miceli et al., 2009, where *KCNQ2*-D212G was reported to moderately impair voltage sensing in KCNQ2/3 heterotetramers [29]. This discrepancy may arise due to differences in experimental conditions. Notably, Miceli et al. included the *KCNQ3*-P574S variant, which could have impacted the behavior of *KCNQ2*-D212G and contributed to the observed phenotype. While the exact effect of *KCNQ3*-P574S on KCNQ2/3 channels remains unknown, this variant on *KCNQ3* may modulate the impact of *KCNQ2*-D212G.

This differential effect between the two mutations aligns with clinical observations and provides insights into how these mutations manifest as distinct clinical phenotypes. During early postnatal development, *KCNQ2* is expressed at high levels, forming predominantly homotetrameric KCNQ2 channels that regulate neuronal excitability and action potential propagation [34]. At this stage, mutations like D212E and D212G have a pronounced effect because KCNQ2 homotetramers are the primary contributors to membrane excitability. As the brain matures, KCNQ3 expression increases, leading to the formation of heterotetrameric KCNQ2/3 channels, which gradually become the dominant KCNQ channels in adult neurons. The *KCNQ2*-D212E mutation has a detrimental effect on both homotetrameric KCNQ2 channels during early development and heterotetrameric KCNQ2/3 channels later in life, consistent with the lifelong and profound impact. In contrast, the *KCNQ2*-D212G mutation primarily affects homotetrameric KCNQ2 channels, causing a milder SLFNE phenotype during early postnatal development. As *KCNQ3* expression increases and heterotetrameric KCNQ2/3 channels become more dominant, the impact of the D212G mutation diminishes, which likely explains why SLFNE symptoms subside with age [29]. Our findings suggest that the *KCNQ2*-D212G mutation has a moderate effect on heterotetrameric KCNQ2/3 channels, helping to explain why SLFNE patients experience seizures early in life that resolve as they age. The timing of *KCNQ3* expression coincides with the subsiding clinical symptoms, suggesting that the formation of KCNQ2/3 heterotetramers mitigates the effects of the *KCNQ2*-D212G mutation.

In conclusion, D212 in the S4-S5 linker is a critical residue for transducing and stabilizing voltage sensor-induced conformational changes (Figure 5). Mutation of this residue, particularly to a slightly larger, charge-conserved glutamate (D212E), results in more severe defects in voltage sensing and gating, consistent with the clinical observations in DEE patients. These findings provide important insights into the biophysical mechanisms underlying the diverse clinical outcomes of *KCNQ2* mutations.

## Materials and methods

### Cell culture and electrophysiological analysis

The human *KCNQ2* (NM_172107.4) and *KCNQ3* (NM_004519.4) genes were purchased from Origene (origine.org) and subcloned into pcDNA3.0 plasmids for amplification and mutagenesis. Introduction of D212E and D212G were generated using the QuikChange site-directed mutagenesis (RNAi core, Academia Sinica, Taiwan), and The sequences of the mutant clones were later confirmed again by an in-house sequencing facility (Sequencing Core, Institute of Biomedical Sciences, Academia Sinica, Taiwan). HEK293 cells were cultured in Dulbecco’s modified Eagle’s medium (DMEM) supplemented with 10% fetal bovine serum, 100 U/mL penicillin, 100 U/mL streptomycin, and 2 mm L-glutamine (Lonza, Walkersville, MD, USA). For transfection, 2 µg of pcDNA3.0 plasmids containing *KCNQ2* or *KCNQ3* genes with 0.2 µg of pN1-eCFP plasmid were introduced into the cells using PolyJet^TM^ (SignaGen® Laboratories). The GFP signal was used to identify successfully transfected cells. Electrophysiological measurements were performed 1–3 days post-transfection [35]. To assess potential morphological changes, the transfected HEK293 cells were examined under a bright-field and fluorescence microscope. No significant morphological alterations were observed 24–48 hours post-transfection, as shown in Supplementary Figure S1.

### Whole-cell patch-clamp analysis

KCNQ currents were recorded using the whole-cell patch-clamp technique with an Axopatch 200B amplifier (Molecular Devices, USA). Cells were bathed in a standard extracellular solution containing 150 mM NaCl, 10 mM HEPES, 5 mM KCl, 2 mM CaCl_2_, 1 mM MgCl_2_, 10 mM glucose and adjusted to pH 7.4 with NaOH. Data were acquired at 10 kHz using pCLAMP software (Molecular Devices, USA). Patch pipettes were pulled from 1.5 mm borosilicate glass capillaries (Sutter Inc, USA) and had resistances of 2–4 MΩ when filled with the intracellular solution containing 135 mM K gluconate, 15 mM KCl, 10 mM HEPES, 5 mM Mg_2_ATP, 1 mM Na_3_GTP, 10 mM sodium phosphocreatine, 5 mM EGTA, and adjusted to pH 7.4 with KOH. A liquid junction potential of −14.2 mV was calculated, and voltages were corrected offline by subtracting the liquid junction potential. Whole-cell access resistances ranged from 5 to 20 MΩ. All experiments were conducted at room temperature (~25°C). The whole-cell input capacitance was directly determined from the amplifier settings. Data acquisition and analysis were performed using Clampex 10.0 software (Molecular Devices, Sunnyvale, CA, USA). All chemicals, unless otherwise stated, were purchased from Sigma-Aldrich (USA). For activation curves, the data were fitted to a Boltzmann function:

(1) $$Y=\;\frac{1}{1+\exp\left(\frac{V-V1/2}{k}\right)}$$

to determine the activation properties, where V represents the voltage, V_1/2_ is the voltage at which 50% of the channels are activated, and *k* is the slope factor.

For activation and deactivation time constants, the traces were fitted to a single exponential equation:

(2) $$Y\;=\;1\;-\;e^{-t/\tau }+\;C$$

where τ represents the time constant. The τ values were plotted against the test voltage pulses to analyze voltage-dependent kinetics.

The maximal conductance density was calculated from the current difference between 55.8 mV and 65.8 mV and was further normalized to the cell capacitance. Data are presented as the mean ± standard deviation (SD). Statistical significance was determined using two-tailed independent t-tests or analysis of variance (ANOVA), with *p* < 0.05 considered significant.

### In silico simulation

The structures of wild-type, D212E, and D212G human KCNQ2 channels were predicted using I-TASSER [36–38] and AlphaFold2. The closed (PDB: 8SIN) and open (PDB: 8SIK) structures of human KCNQ1 were used as templates for *de novo* structure prediction of KCNQ2 [13]. Predicted models were generated and visualized using PyMOL3.0.3.

## Supplementary Material

Supplemental Material

Suppl fig 1.tiff

## Data Availability

The data supporting this study’s findings are available from the corresponding author, S.B. Yang, upon reasonable request.
