# Supplementary material for: Biophysical and structural mechanisms of epilepsy-associated mutations in the S4-S5 Linker of KCNQ2 channels
Source: Channels (Austin). 2025 Feb 19;19(1):2464735. doi: 10.1080/19336950.2025.2464735 (PMC11845087; doi:10.1080/19336950.2025.2464735)
Supplement: Supplemental Material [file KCHL_A_2464735_SM4221.docx]

**Supplemental Figure 1: Bright-field and fluorescence micrographs of HEK293 cells transfected with KCNQ2 WT, KCNQ2-D212E, KCNQ2-D212G, or KCNQ2/3.** Bright-field images (left) and corresponding GFP fluorescence micrographs (middle) are shown alongside merged images (right). No abnormal morphology was observed in cells transfected with any of the constructs**.**

**Supplemental Figure 1 Alt-text**

Bright-field and fluorescence micrographs of HEK293 cells transfected with KCNQ2 WT, KCNQ2-D212E, KCNQ2-D212G, or KCNQ2/3. No abnormal cell morphology was observed for any construct.
