# Supplementary figures and images for: Biophysical and structural mechanisms of epilepsy-associated mutations in the S4-S5 Linker of KCNQ2 channels
Source: Channels (Austin). 2025 Feb 19;19(1):2464735. doi: 10.1080/19336950.2025.2464735 (PMC11845087; doi:10.1080/19336950.2025.2464735)

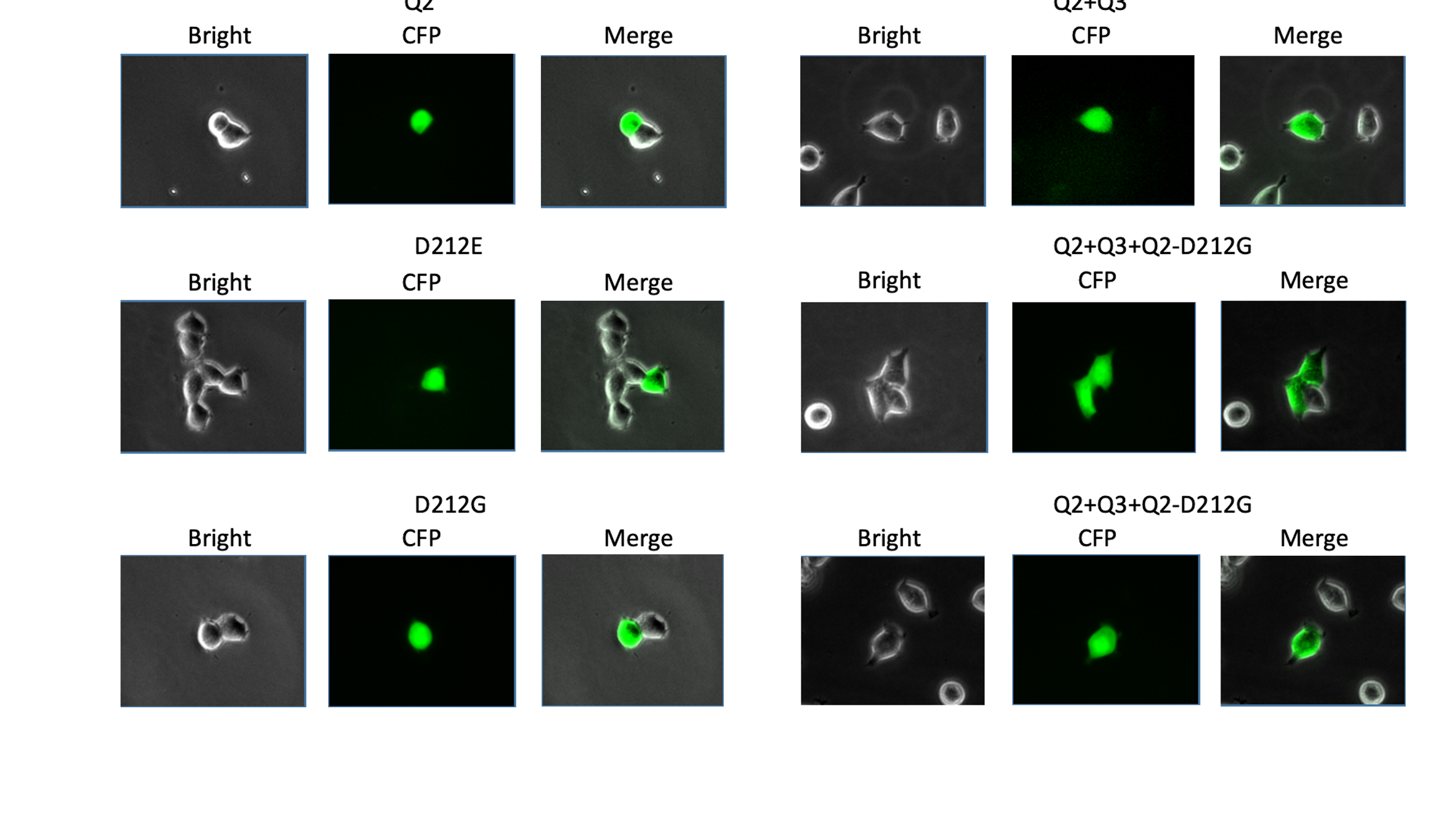

Supplement: Suppl fig 1.tiff [file KCHL_A_2464735_SM8823.tiff]
